# Effects of Exergame-Based Interventions on Executive Functions and Motor Skills in Children with Autism Spectrum Disorder: A Systematic Review

**DOI:** 10.3390/sports14050174

**Published:** 2026-04-28

**Authors:** Noelia Vigil-Torres, María del Carmen Carcelén-Fraile, Teresa Martínez-Redecillas, Daniela Cecic-Mladinic

**Affiliations:** 1 Department of Educational Sciences, Faculty of Social Sciences, University of Atlántico Medio, 35017 Las Palmas de Gran Canaria, Spain; noelia.vigil@pdi.atlanticomedio.es (N.V.-T.); daniela.cecic@pdi.atlanticomedio.es (D.C.-M.); 2 International Scientific Association on Innovation in Education and Health (ACIINES), 23007 Jaén, Spain; 3 International Network of Educational Law, 35017 Las Palmas de Gran Canaria, Spain; 4 Faculty of English Philology, University of Granada, 18012 Granada, Spain; tmartinezredecillas@gmail.com

**Keywords:** autism spectrum disorder, exergames, executive functions, motor development, physical activity, systematic review

## Abstract

Children with Autism Spectrum Disorder (ASD) frequently present impairments in executive functions and motor skills, which can negatively affect academic performance, adaptive behavior, and daily functioning. Exergames have emerged as a potentially engaging cognitive–motor intervention. The objective of this systematic review was to analyze the effects of exergame-based interventions on executive function components (particularly inhibitory control and cognitive flexibility) and motor skills in children with ASD. A systematic review was conducted in accordance with PRISMA guidelines, with the protocol registered in PROSPERO. Electronic searches were performed in PubMed, Scopus, Web of Science, and ERIC. Intervention studies published within the last five years and assessing exergame-based interventions in children with ASD were included. Methodological quality was evaluated using the PEDro scale. Six studies met the inclusion criteria. Exergame-based interventions were associated with improvements in executive functions, particularly inhibitory control (reported in two studies using Stroop- and Flanker-type tasks) and cognitive flexibility (assessed in two studies using the Wisconsin Card Sorting Test), although results varied depending on intervention duration and design. Acute interventions (single-session) primarily influenced inhibitory control, whereas longer-term programs showed broader cognitive and motor adaptations. Improvements in motor outcomes, including gross motor development, coordination, and fundamental motor skills, were reported in four studies. Methodological quality ranged from 4 to 6 points on the PEDro scale, indicating fair to good quality. Considerable heterogeneity was observed in intervention protocols, duration, and outcome measures. Exergame-based interventions may represent a potentially promising approach for targeting executive functions and motor skills in children with ASD; however, the current evidence is limited and heterogeneous. Not all included studies assessed both cognitive and motor outcomes, and findings should therefore be interpreted with caution. Further high-quality randomized controlled trials are needed to confirm these effects and establish optimal intervention parameters.

## 1. Introduction

Autism Spectrum Disorder (ASD) is a neurodevelopmental condition characterized by persistent deficits in social communication and interaction, as well as restricted and repetitive patterns of behavior [1]. The prevalence of ASD has increased considerably over recent decades, highlighting the importance of identifying effective therapeutic and educational interventions aimed at improving functional outcomes and quality of life in affected individuals [2]. Beyond the core diagnostic symptoms, children with ASD frequently present deficits in motor development and executive functioning [3], which can substantially interfere with academic performance, adaptive behavior, and participation in daily activities [4].

Motor impairments are highly prevalent in children with ASD and may include deficits in coordination, balance, locomotor abilities, and object control skills [5]. These difficulties can limit engagement in physical activity and social play, both of which are essential for childhood development [6]. Furthermore, emerging evidence suggests a strong relationship between motor development and cognitive functioning [7]. Motor activity requires continuous integration of sensory processing, motor planning, and cognitive control, indicating that improvements in motor competence may support cognitive development [8].

Executive functions represent a group of higher-order cognitive processes responsible for goal-directed behavior and adaptive functioning [9]. These processes include inhibitory control, cognitive flexibility, working memory, and attentional regulation [10]. Children with ASD often demonstrate executive function impairments, which are associated with difficulties in behavioral regulation, emotional control, and learning performance [11]. Consequently, interventions targeting executive functioning have gained increasing interest as potential strategies to improve functional independence and social adaptation in this population [12].

Physical activity has been widely investigated as a therapeutic approach capable of promoting both motor and cognitive improvements in children with neurodevelopmental disorders [13]. Exercise has been associated with neurophysiological adaptations, including enhanced neural connectivity, increased cerebral blood flow, and modulation of neurotransmitter systems linked to cognitive processing [14]. However, maintaining motivation and adherence to conventional exercise programs remains a significant challenge for children with ASD due to limited interest, sensory sensitivities, and behavioral difficulties [15].

In recent years, technological advancements have introduced innovative rehabilitation and training approaches that integrate physical activity with interactive digital environments [16]. Exergames, defined as video games that require body movement to interact with virtual scenarios, have emerged as a promising intervention tool [17]. These systems combine motor engagement with continuous cognitive demands, including attentional processing, decision-making, response inhibition, and problem-solving [18]. The interactive and rewarding nature of exergames may enhance engagement and adherence to intervention programs, representing an advantage over traditional therapeutic approaches [19].

Theoretical models of cognitive–motor interaction suggest that simultaneous activation of motor and executive control networks may promote neuroplastic adaptations supporting improvements in both domains [20]. Exergames typically require individuals to process multisensory stimuli, adjust movement strategies in real time, and respond to dynamic environmental feedback. These characteristics make exergames particularly relevant as training tools capable of stimulating executive functions during physically active tasks [21].

Although previous research has explored the effects of physical activity and digital-based interventions in children with neurodevelopmental disorders, important limitations remain in the literature regarding exergame-based interventions in children with ASD. Several previous reviews have examined the effects of exergames or physical activity programs in this population; however, these reviews have often reported heterogeneous cognitive outcomes without distinguishing between specific executive function components, limiting the identification of domain-specific effects [22,23]. In particular, key executive processes such as inhibitory control and cognitive flexibility, both of which are strongly related to behavioral regulation and adaptive functioning, have not been systematically analyzed within exergame-based interventions. Similarly, motor outcomes have frequently been described in general terms, without clearly linking improvements to specific domains such as coordination, locomotor skills, or object control. In addition, previous reviews have largely focused on physical or general cognitive outcomes, with limited emphasis on the interaction between executive functions and motor performance within a cognitive–motor framework. This lack of integration restricts understanding of how exergame-based interventions may simultaneously influence cognitive and motor development. Furthermore, earlier reviews were conducted when the number of available experimental and randomized studies was still limited. More recent studies have incorporated structured exergame protocols, advanced technologies (e.g., virtual and augmented reality), and more refined outcome measures. This evolution highlights the need for an updated and more focused synthesis of the evidence.

Therefore, the present systematic review aims to analyze the effects of exergame-based interventions on executive function components (particularly inhibitory control and cognitive flexibility) and motor skills (including coordination, gross motor development, and fundamental motor skills) in children diagnosed with ASD. In addition to providing an updated synthesis, the present review contributes to the literature in three ways. First, it focuses on inhibitory control and cognitive flexibility as distinct executive function domains, rather than treating cognition as a broad or undifferentiated construct. Second, it examines these executive outcomes alongside motor skills within a cognitive–motor framework, allowing for a more integrated interpretation of exergame-related effects. Third, it synthesizes recent intervention studies incorporating emerging exergame technologies and more refined outcome measures that were not represented in earlier reviews.

## 2. Materials and Methods

### 2.1. Information Sources

A systematic review was conducted to examine the effectiveness of exergame-based interventions on motor skills and executive functions in children with ASD. The review was designed following internationally recognized methodological standards for systematic reviews. The study aimed to identify, analyze, and synthesize evidence from intervention studies investigating the impact of exergame-based interventions on motor and cognitive outcomes in the pediatric ASD population.

The protocol of this systematic review was registered in the International Prospective Register of Systematic Reviews (PROSPERO) prior to data extraction to ensure transparency and methodological rigor. The registration code was CRD420261305764. The reporting of this systematic review was conducted in accordance with the Preferred Reporting Items for Systematic Reviews and Meta-Analyses (PRISMA) guidelines [24]. The PRISMA checklist is also available in the Supplementary Materials section.

### 2.2. Search Strategy

The literature search was conducted in February 2026. Different keywords were combined using Boolean operators in the following search string: (“exergame” OR “active video game” OR “virtual reality” OR “interactive video game” OR “motion-based video game”) AND (“autism” OR “autism spectrum disorder” OR “ASD”) AND (“motor skills” OR “motor development” OR “motor performance” OR “executive function” OR “cognitive function”).

The search strategy was adapted to each of the consulted databases, including PubMed, Scopus, Web of Science (WoS), and ERIC, while maintaining a consistent combination of keywords and Boolean operators across databases to ensure comparability of the search process. Filters were applied to limit the results to studies published within the last five years and involving human participants within the pediatric age range. In addition, a manual search was performed by reviewing the reference lists of the included studies to identify potentially relevant articles not retrieved during the electronic search.

### 2.3. Eligibility Criteria

Studies were selected according to predefined inclusion and exclusion criteria. Articles were included if they investigated the effects of exergame-based interventions in children with a formal diagnosis of Autism Spectrum Disorder, based on established clinical or diagnostic criteria. Eligible studies were required to assess outcomes related to specific executive function components (e.g., inhibitory control, cognitive flexibility, or related processes) and/or motor performance (e.g., coordination, gross motor development, or fundamental motor skills). Intervention studies evaluating exergame-based interventions were included. Both randomized and non-randomized experimental designs were considered. In addition, single-group pre–post intervention studies were also included, provided that they evaluated the effects of exergame-based interventions on executive function and/or motor outcomes. Comparator conditions, when present, included conventional therapy, traditional physical activity, sedentary activities, or no additional intervention.

Only studies published within the last five years were included. This restriction was applied due to the rapid evolution of exergame technologies, including advances in motion-tracking systems, virtual and augmented reality, and interactive platforms, which may limit the applicability of older studies to current intervention contexts. This criterion was also intended to prioritize interventions using platforms, protocols, and outcome measures more representative of current practice. However, this restriction may have excluded earlier high-quality studies, which should be considered a limitation when interpreting the scope of the evidence. No language restrictions were applied during the search and selection process.

Studies were excluded if participants did not have a formal diagnosis of ASD or if results for participants with ASD were not reported separately from other developmental disorders. Reviews, meta-analyses, conference abstracts, protocols, editorials, and qualitative studies were also excluded. Additionally, studies that did not include exergame-based interventions or did not assess motor or executive function outcomes were excluded.

### 2.4. Study Selection Process

The study selection process began with the removal of duplicate records. Subsequently, titles and abstracts were screened to exclude studies that did not meet the predefined eligibility criteria. Articles that met the initial criteria were retrieved and assessed in full-text format to determine their suitability for inclusion in the systematic review. The study selection process (screening of titles/abstracts and full-text assessment) was conducted independently by two reviewers. Any disagreements were resolved through discussion and consensus. A total of six studies met the inclusion criteria and were included in the final qualitative synthesis.

### 2.5. Data Extraction

Data extraction was conducted using a standardized data collection form developed for this review. Relevant information was extracted from each included study, including authors, year of publication, country, sample size, participant characteristics (age and gender), intervention characteristics, and outcome measures. Details regarding the exergame interventions were also collected, including type of exergame, duration of the intervention, frequency of sessions, and total intervention period. Additionally, information related to the assessment tools used to evaluate motor skills and executive functions was extracted. Data extraction was performed independently by two reviewers, and both reviewers were blinded to each other’s initial assessments during the process. Any discrepancies were resolved through discussion and consensus. It was not necessary to contact the study authors to obtain missing or unclear data. When studies reported incomplete statistical information, all available descriptive and inferential data were extracted as reported by the original authors. A formal assessment of publication bias was not conducted due to the small number of included studies and the heterogeneity of the data. A meta-analysis was not conducted due to substantial heterogeneity across studies in intervention duration, exergame platforms, outcome measures, and study designs, which limited the comparability of quantitative findings and supported the use of a narrative synthesis approach.

### 2.6. Quality Assessment

The methodological quality of the included studies was evaluated using the Physiotherapy Evidence Database (PEDro) scale [25]. The PEDro scale is a validated tool designed to assess the methodological quality and internal validity of experimental studies, particularly in physiotherapy and rehabilitation research. The PEDro scale consists of 11 items evaluating aspects such as random allocation, concealed allocation, baseline comparability, blinding procedures, follow-up, intention-to-treat analysis, and statistical reporting. The first item relates to external validity and is not included in the total score. The total PEDro score ranges from 0 to 10, with higher scores indicating better methodological quality. PEDro scores were interpreted as follows: 9–10 excellent quality, 6–8 good quality, 4–5 fair quality, and below 4 poor quality [26]. Quality assessment was performed independently by two reviewers. Any disagreements were resolved through discussion until consensus was reached. The methodological quality scores of the included studies were used to support the interpretation of the findings.

## 3. Results

### 3.1. Study Selection Process

**Figure 1:**
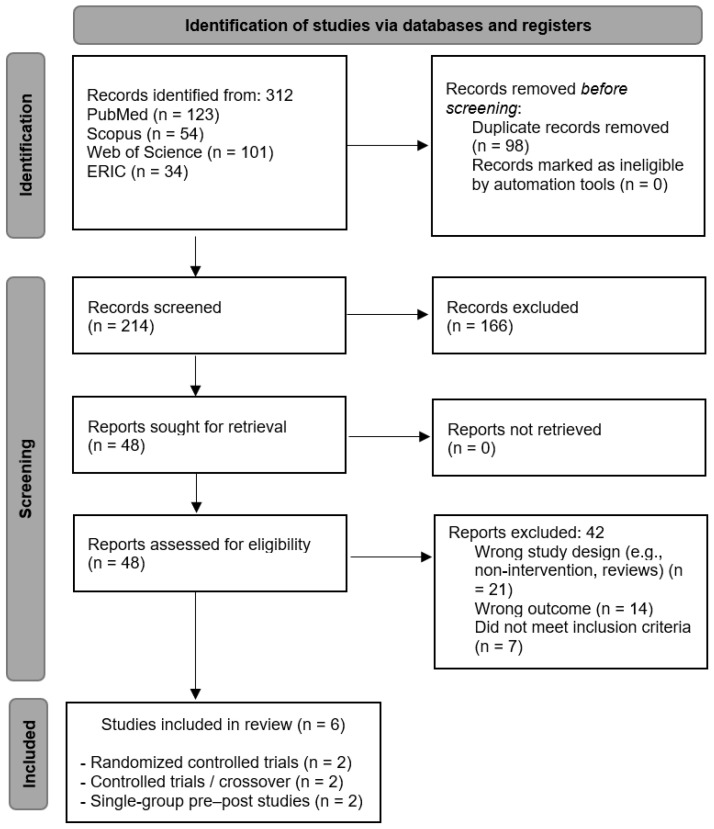
Study selection process flow chart.

A total of 312 records were identified through database searches, including PubMed (*n* = 123), Scopus (*n* = 54), Web of Science (*n* = 101), and ERIC (*n* = 34). After removing duplicates (*n* = 98), 214 records remained for screening. Following title and abstract screening, 166 records were excluded, and 48 full-text articles were assessed for eligibility. Of these, 42 studies were excluded because of inappropriate study design (*n* = 21), outcomes unrelated to motor skills or executive functions (*n* = 14), or failure to meet inclusion criteria (*n* = 7). Six studies met all eligibility criteria and were included in the qualitative synthesis. The study selection process is shown in Figure 1, following PRISMA 2020 guidelines.

### 3.2. Methodological Quality

The methodological quality of the included studies was assessed using the PEDro scale. Overall, the methodological quality of the studies ranged from fair to good, with PEDro scores between 4 and 6 out of 10. Two studies demonstrated good methodological quality, achieving PEDro scores of 6/10 [27,28]. These studies included random allocation, baseline comparability between groups, adequate follow-up, and statistical comparisons between intervention groups. Additionally, both studies reported point estimates and measures of variability, which strengthen the reliability of their findings. Two studies demonstrated moderate methodological quality, with PEDro scores of 5/10 [29,30]. These studies incorporated randomized or controlled intervention designs and reported appropriate statistical analyses. However, limitations were observed in allocation concealment and blinding procedures, which may increase the risk of bias. The remaining two studies showed fair methodological quality, achieving PEDro scores of 4/10 [31,32]. These studies lacked randomization, intention-to-treat analysis, and between-group comparisons, mainly due to pre–post or single-group study designs. Across all included studies, blinding of participants, therapists, and assessors was rarely reported. This limitation is common in exercise and rehabilitation interventions, where blinding procedures are often difficult to implement. Nevertheless, most studies clearly described eligibility criteria, intervention protocols, and outcome measures. A summary of methodological quality scores for each study is presented in Table 1.

### 3.3. Study Characteristics

Six studies published within the last five years were included in this systematic review. These studies were conducted in five countries (Brazil, Iran, South Korea, Portugal, and Spain), reflecting growing international interest in exergame-based interventions for children with ASD [27,28,29,30,31,32]. Sample sizes ranged from 5 to 52 participants, and most studies included small to moderate samples. Participants were generally aged 6–18 years, with a predominance of males, consistent with the higher prevalence of ASD in boys. Study designs included two randomized controlled trials [27,29], one controlled trial [28], one crossover study [30], and two single-group pre–post studies [31,32]. When included, control conditions involved conventional therapy, traditional physical activity, sedentary activities, or no additional intervention.

Exergame platforms varied across studies. Three studies used Xbox Kinect systems [27,30,31], one used Nintendo Wii [32], and two implemented technology-enhanced systems, including augmented reality or ICT-based exergames [28,29]. These differences reflect variation in technological complexity and cognitive–motor demands. Intervention duration ranged from a single acute session [30] to longer-term programs lasting 4–12 weeks [27,28,29,31], with one study extending to 12 months [32]. Session frequency ranged from one to three sessions per week, most commonly two, while session duration varied between 15 and 60 min. Overall, interventions were generally structured and repeated training programs combining motor engagement with varying cognitive demands. Commercial platforms such as Kinect and Wii mainly emphasized whole-body movement and coordination, whereas AR- and ICT-based systems incorporated more explicit cognitive challenges, including decision-making, response inhibition, and task switching. Detailed study characteristics are presented in Table 2.

### 3.4. Study Results

Across the six included studies, exergame-based interventions combined motor activity with concurrent cognitive demands, including attentional control, response inhibition, and task switching. Given the heterogeneity in study design, intervention protocols, and outcome measures, findings were synthesized narratively. Because not all studies assessed both executive function and motor outcomes, results are presented separately by outcome domain. For clarity, findings are also described according to intervention duration, distinguishing between acute (single-session) and longer-term interventions.

#### 3.4.1. Executive Functions and Cognitive Outcomes

Executive function outcomes were reported in three of the six included studies (50%), primarily focusing on inhibitory control and cognitive flexibility. Overall, all three studies reported improvements in at least one executive function outcome, although the magnitude and consistency of effects varied depending on the specific component and measurement approach.

Cognitive flexibility (WCST). Two studies assessed cognitive flexibility using the Wisconsin Card Sorting Test. Both reported improved performance following exergame-based interventions. Rafiei Milajerdi et al. [27] reported significant improvements over time in conceptual responses (F(1,53) = 10.61, *p* < 0.01, partial η^2^ = 0.16) and reductions in perseverative errors (F(1,53) = 14.31, *p* < 0.01, partial η^2^ = 0.21), indicating improved cognitive flexibility. In addition, a significant group effect was observed for correct responses (F(2,53) = 5.43, *p* < 0.01, partial η^2^ = 0.17), with post hoc analyses showing better performance in the Kinect group compared with the control and SPARK groups. However, no significant group × time interaction was observed for WCST outcomes. Nekar et al. [29] also reported significant improvements in cognitive flexibility. Reaction time improved from 1102.08 ± 5.46 ms to 1092.25 ± 5.15 ms in the intervention group, compared with a smaller improvement in the control group (1102.00 ± 5.20 ms to 1098.58 ± 5.12 ms), with a significant time × group interaction (F = 7.184, *p* = 0.016). Response accuracy increased from 79.08 ± 3.02 to 84.83 ± 2.40 points in the intervention group compared with 79.25 ± 4.11 to 81.92 ± 2.42 points in the control group (F = 6.349, *p* = 0.043). Together, these findings indicate a consistent positive pattern for cognitive flexibility.

Inhibitory control (Stroop/Flanker). Two studies assessed inhibitory control using Stroop- and Flanker-type tasks. Nekar et al. [29] reported significant improvements in cognitive inhibition performance. Reaction time improved from 1104.92 ± 8.17 ms to 1102.17 ± 7.34 ms in the intervention group, whereas the control group showed minimal change (1104.83 ± 5.13 ms to 1104.25 ± 5.17 ms), with a significant time × group interaction (F = 5.200, *p* = 0.045). Response accuracy also increased from 76.08 ± 2.06 to 84.33 ± 2.10 points in the intervention group compared with 76.33 ± 3.79 to 81.08 ± 2.42 points in controls (F = 4.351, *p* = 0.042). Miranda et al. [30], an acute intervention study, found significant improvements specifically in incongruent Flanker reaction time following a single exergame session (849 ± 270 ms) compared with both the sedentary control session (969 ± 295 ms; mean difference = −120 ms, 95% CI −202 to −38; *p* = 0.01; Cohen’s d = −1.1) and the traditional games session (938 ± 330 ms; mean difference = −89 ms, 95% CI −159 to −19; *p* = 0.02; Cohen’s d = −1.0). A significant overall condition effect was observed for incongruent reaction time (*p* = 0.02; η^2^ = 0.38). However, no statistically significant effects were found for congruent reaction time or for congruent and incongruent accuracy outcomes (*p* > 0.05).

Working memory. Nekar et al. [29] also reported significant improvements in working memory scores in the intervention group (*p* = 0.032). However, the time × group interaction was not statistically significant (F = 0.016, *p* = 0.977), suggesting that improvements should be interpreted cautiously.

Differences also emerged according to intervention duration. The acute intervention study [30] produced immediate improvements primarily in inhibitory-control reaction time, whereas longer-term interventions [27,29] (4–8 weeks) reported broader improvements across cognitive flexibility, inhibitory control, and working memory outcomes. Overall, the evidence points to a positive pattern of change in executive function outcomes, although effect magnitude remains difficult to compare because of variability in measures and reporting methods.

#### 3.4.2. Effects on Motor Skills

Motor outcomes were assessed in four of the six included studies (67%), primarily using standardized measures of motor development and coordination. All four studies reported gains in at least one motor outcome following exergame-based interventions, indicating a generally positive pattern across studies. Rafiei Milajerdi et al. [27] found a significant group × time interaction for aiming and catching skills measured with the MABC-2 (F(2,53) = 4.12, *p* = 0.02, partial η^2^ = 0.13), with the SPARK group improving from 2.85 ± 2.37 to 4.45 ± 2.64 points. In contrast, no significant improvements were observed for manual dexterity (F(2,53) = 0.47, *p* = 0.63, partial η^2^ = 0.02) or balance (F(2,53) = 0.80, *p* = 0.50, partial η^2^ = 0.03). Kwon et al. [28] reported significant improvements in muscular strength and fundamental motor skills following the exergame intervention. Standing long jump performance improved from 88.3 ± 24.9 cm to 98.8 ± 29.3 cm (F(1,50) = 18.79, *p* < 0.001). Hop skill scores increased from 3.20 ± 1.70 to 5.08 ± 2.02 (F(1,50) = 21.71, *p* < 0.001). Significant improvements were also observed in overhand throw (F(1,50) = 27.94, *p* < 0.001) and dribbling performance (F(1,50) = 12.95, *p* < 0.001). However, horizontal jump performance showed only a non-significant improvement from 4.96 ± 2.11 to 5.76 ± 1.82 (F(1,50) = 3.39, *p* = 0.07). Grola et al. [31] reported significant improvements in gross motor development following a 12-week exergame-based physiotherapy intervention. Gross Motor Quotient scores increased from a median of 52 to 67 (*p* = 0.0277). Significant improvements were also observed in the locomotor subtest, which increased from a median of 0 to 66 (*p* = 0.0277), and in object control performance, which improved from a median of 36 to 48 (*p* = 0.0180). However, no statistically significant improvements were identified at the level of individual TGMD-2 motor skills. Oliveira Neves et al. [32] also reported improvements in motor performance following a non-immersive virtual reality exergame intervention. General Motor Age increased in all participants, for example from 85.9 to 106.6 months in one participant, from 57.6 to 82.43 months in another participant, and from 98 to 110 months in another participant. Improvements were also observed across fine motor skills, global motor skills, balance, body schema, spatial organization, and temporal organization. However, the study reported only descriptive pre–post changes without inferential statistical analyses. Compared with executive outcomes, findings for motor skills were somewhat more consistent across studies. Motor benefits were mainly reported in longer-term interventions (≥4 weeks), particularly in coordination, locomotor skills, and overall motor development. Acute interventions did not assess motor outcomes, limiting conclusions regarding short-term effects.

Overall, the evidence supports a positive pattern of change in motor outcomes, although effect magnitude remains difficult to compare because of differences in assessment tools, reporting methods, and intervention protocols.

## 4. Discussion

This systematic review examined exergame-based interventions in children with ASD, focusing on executive function and motor outcomes. Overall, the included studies suggest improvements in inhibitory control, cognitive flexibility, and motor performance, although these findings should be interpreted cautiously due to the small number of studies, heterogeneity in interventions and outcome measures, and moderate methodological quality (PEDro scores: 4–6/10). The most consistent improvements were observed in motor performance, while executive function improvements were most frequently reported for inhibitory control and cognitive flexibility. However, evidence for other executive domains remains limited due to the lower number of studies assessing these outcomes.

Across the included studies, executive outcomes were reported most frequently for inhibitory control (Stroop/Flanker-type tasks) and cognitive flexibility (WCST). Many exergame tasks involve rapid responses, attentional engagement, and response inhibition, which may explain the relatively more consistent improvements observed in inhibitory control. Acute improvements in inhibitory-control reaction time observed in single-session studies may reflect short-term changes in attentional engagement or task-related activation, consistent with broader exercise–cognition literature. Improvements in reaction time without corresponding changes in accuracy may also reflect changes in processing efficiency or speed–accuracy trade-offs. For cognitive flexibility, improvements in WCST outcomes may be related to repeated exposure to rule-based and feedback-driven tasks, although this interpretation remains tentative given the limited number of studies and methodological variability. In contrast, working memory and planning were less frequently assessed, preventing stronger conclusions regarding these executive domains.

Regarding motor outcomes, several studies reported improvements in coordination, locomotor performance, object control skills, and overall motor development following exergame interventions. These findings may be explained by the repetitive practice of movement patterns, real-time feedback, and high levels of engagement typically associated with exergaming. However, variability in intervention duration, platform type, and training intensity makes it difficult to determine which specific intervention characteristics are most effective.

Our findings build on previous reviews examining exergames in children with ASD. Earlier reviews, such as Fang et al. [33], reported improvements in cognitive and physical outcomes but were limited by the small number of intervention studies and the use of heterogeneous cognitive measures without detailed analysis of specific executive function domains. Similarly, Lima et al. [34] highlighted possible benefits for physical fitness, cognitive performance, and behavioral symptoms while emphasizing the emerging nature of the evidence base. The present review extends previous literature by providing an updated synthesis of recent intervention studies and by specifically examining inhibitory control and cognitive flexibility, two executive domains that have received limited focused attention in previous reviews. From a practical perspective, these findings suggest that exergame-based interventions may represent a promising complementary strategy for professionals working with children with ASD, including educators, therapists, and clinicians. Their interactive and motivating nature may help increase participation in physical activity while simultaneously stimulating cognitive processes. However, given the current limitations of the evidence base, exergames should be considered a complementary tool rather than a standalone intervention.

Some previous studies (e.g., Hilton et al. [35]) have reported associations between executive function and motor performance in children with ASD. Although exergame-based interventions inherently combine cognitive and motor demands, current evidence does not allow for firm conclusions regarding whether improvements in both domains occur through shared cognitive–motor mechanisms. Not all included studies assessed both domains simultaneously, and the included studies were not specifically designed to test these mechanisms directly [36]. Accordingly, interpretations related to cognitive–motor integration remain hypothesis-generating rather than confirmatory.

Several limitations should be considered. First, the small number of included studies (*n* = 6) and substantial heterogeneity in intervention protocols, duration, exergame platforms, and outcome measures limit generalizability and comparability. Second, methodological limitations, including small sample sizes, limited blinding, and lack of allocation concealment, may increase risk of bias. Variability in executive function assessments also complicates interpretation of cognitive outcomes. The use of the PEDro scale presents limitations when applied to heterogeneous or non-randomized designs and was therefore interpreted alongside study design characteristics. Additional limitations include the predominance of short- to medium-term outcomes, the absence of quantitative synthesis, and the lack of formal assessment of publication bias. Future studies should adopt more rigorous experimental designs, standardized outcome measures, and longer follow-up periods to better clarify the effects of exergame interventions in children with ASD.

## 5. Conclusions

The findings of this systematic review suggest that exergame-based interventions represent a promising approach for improving specific executive function components and motor skills in children with ASD, although the evidence remains limited by the small number of studies, methodological variability, and moderate study quality. Current evidence indicates positive effects particularly for inhibitory control, and to a lesser extent cognitive flexibility, as well as improvements in motor coordination and gross motor development. However, because not all included studies assessed both cognitive and motor outcomes, conclusions regarding simultaneous cognitive–motor effects remain limited. From a practical perspective, exergame-based interventions may be considered a complementary tool to support cognitive–motor training in educational or therapeutic contexts, particularly as an engaging strategy to promote participation and structured practice. However, they should currently be viewed as an adjunct rather than a replacement for established interventions, given the limited and heterogeneous evidence base. Future research should prioritize well-designed randomized controlled trials with larger samples, standardized intervention protocols, and long-term follow-up. Further studies are also needed to clarify potential neurocognitive mechanisms and determine optimal training parameters. Overall, exergames appear to be a feasible intervention approach with potential benefits, although more robust and consistent evidence is required before definitive conclusions can be established.

## Figures and Tables

**Table 1:** Methodological quality of the included articles.

Study	1	2	3	4	5	6	7	8	9	10	11	Total (0–10)
Rafiei Milajerdi et al. [27]	1	1	0	1	0	0	0	1	1	1	1	6
Kwon et al. [28]	1	1	0	1	0	0	0	1	1	1	1	6
Nekar et al. [29]	1	1	0	1	0	0	0	1	0	1	1	5
Miranda et al. [30]	1	1	0	0	0	0	0	1	1	1	1	5
Grola et al. [31]	1	1	0	1	0	0	0	0	0	1	1	4
Oliveira Neves et al. [32]	1	1	0	1	0	0	0	0	0	1	1	4

Scoring criteria: (1) eligibility specified; (2) random allocation; (3) concealed allocation; (4) comparable groups at baseline; (5) participant blinding; (6) therapist blinding; (7) assessor blinding; (8) adequate follow-up; (9) intention-to-treat analysis; (10) between-group statistical comparisons. Each item is scored as Yes = 1 or No = 0.

**Table 2:** Characteristics of the included studies.

Study and Year	Study Design	Characteristics of the Participants		Characteristics of the Intervention		Control	Outcomes
		Sample	Age	Platform Type	Session Duration, Frequency		
Rafiei Milajerdi, 2021 [27]	RCT	37 (17/20)	6–10	Exergame (Xbox Kinect tennis)	35 min 3 sessions/8 weeks	Non-exergaming	Cognitive flexibility (WCST)
Kwon et al., 2022 [28]	Controlled trial	52 (27/25)	10.2 (mean)	ICT-based exergame	60 min 2 sessions/12 weeks	Traditional physical activity	Fundamental motor skills; Physical fitness
Nekar et al., 2022 [29]	RCT	24 (12/12)	6–18	AR-based exergame (UINCARE)	15 min 2 session/4 weeks	Non-exergaming	Cognitive flexibility (WCST); Inhibition control (Stroop)
Miranda et al., 2025 [30]	Crossover	9	8.6 (mean)	Exergame (Just Dance, Kinect)	20 min 1 session	Active traditional games	Inhibitory control (Flanker); Motor coordination (KTK); Cognitive ability (Raven)
Grola et al., 2025 [31]	Single-group pre–post	9	7–10	Exergame (Xbox Kinect)	30 min 2 session/12 weeks	Usual physical activity	Gross motor development (TGMD-2); ASD severity (CARS)
Oliveira Neves et al., 2025 [32]	Single-group pre–post	5	7–11	Exergame (Nintendo Wii)	60 min 1 session/12 weeks	Usual physical activity	Motor development (MDS)

N = total number of participants; values in parentheses indicate experimental/control group distribution. WCST = Wisconsin Card Sorting Test; TGMD-2 = Test of Gross Motor Development (2nd edition); CARS = Childhood Autism Rating Scale; KTK = Körperkoordinationstest für Kinder; MDS = Motor Development Scale.

## Data Availability

Not applicable.

## Supplementary Materials

The following supporting information can be downloaded at: https://www.mdpi.com/article/10.3390/sports14050174/s1. PRISMA 2020 Checklist.

## Abbreviations

The following abbreviations are used in this manuscript:

ASD	Autism Spectrum Disorder
PRISMA	Preferred Reporting Items for Systematic Reviews and Meta-Analyses
PROSPERO	International Prospective Register of Systematic Reviews
PEDro	Physiotherapy Evidence Database
WCST	Wisconsin Card Sorting Test
TGMD-2	Test of Gross Motor Development, Second Edition
CARS	Childhood Autism Rating Scale
KTK	Körperkoordinationstest für Kinder
MDS	Motor Development Scale
ICT	Information and Communication Technology
AR	Augmented Reality
WoS	Web of Science
ERIC	Education Resources Information Center
